# Liver Cancer Stem Cells

**DOI:** 10.4061/2011/486954

**Published:** 2011-06-30

**Authors:** Sameh Mikhail, Aiwu Ruth He

**Affiliations:** Lombardi Comprehensive Cancer Center, Georgetown University Hospital, 3800 Reservoir Road Northwest, Washington, DC 20007, USA

## Abstract

Hepatocellular carcinoma is the most common primary malignancy of the liver in adults. It is also the fifth most common solid cancer worldwide and the third leading cause of cancer-related death. Recent research supports that liver cancer is a disease of adult stem cells. From the models of experimental hepatocarcinogenesis, there may be at least three distinct cell lineages with progenitor properties susceptible to neoplastic transformation. Identification of specific cell surface markers for each of the liver cell types, production of corresponding monoclonal antibodies and cell sorting techniques have together revolutionized the characteristics of normal stem cells. In hepatocarcinogenesis, multiple signaling transduction pathways, important for stem cell proliferation and differentiations, are deregulated. Strategies are being developed to identify and characterize the liver cancer stem cells. Targeting liver cancer stem cells may bring hope to curing hepatocellular carcinoma.

## 1. Introduction

Hepatocellular carcinoma (HCC) is the most common primary malignancy of the liver in adults. It is also the fifth most common solid cancer worldwide and the third leading cause of cancer related death [1, 2]. Moreover, HCC incidence and death rate are rising in the United States and demonstrate the highest annual percent increase of the top 15 cancers by incidence [3]. The worldwide incidence of HCC varies according to the prevalence of hepatitis B (HBV) and hepatitis C (HCV) infection and the age-standardized incidence rates vary from 4.9 per 100,000 population in North America to 80 per 100,000 population in China [4]. It is worth noting that chronic HCV infection is the leading cause of HCC in Europe, Japan, and the United States whereas HBV infection is the leading cause in the majority of Asian and African countries [2]. 

Recent research supports that cancer is a disease of adult stem cells (SC). Adult stem cells are the only cells that persist in the tissue for a sufficient length of time to acquire the requisite number of genetic changes for neoplastic development. In contrast to intestinal mucosa and epidermis where a steady flux of cells occurs from the stem cell zone to the terminally differentiated cells that are imminently to be lost, liver normally exhibits a very low level of cell turnover. However, when abnormal hepatocyte loss occurs, such as after partial hepatectomy (PH) or toxic injury, the liver demonstrates an enormous regenerative capacity. The clonality of HCC is now well established based upon the studies examining viral integration sites of HBV in tumor samples [5], as well as on the determination of restriction fragment length polymorphisms of X-linked genes in tumor cells [6]. However, the cell type that has given rise to HCC has not been universally accepted. From the models of experimental hepatocarcinogenesis, there may be at least three distinct cell lineages with progenitor properties susceptible to neoplastic transformation [7].

## 2. The Stem Cell Origin of Liver Cancer

### 2.1. Hepatocytes Have “Stem Cell” Properties

Hepatocytes in normal adult liver have a lifespan of over a year. However, in response to parenchymal cell loss, the hepatocytes restore the liver mass by self-replication. In rodents, the liver can restore its original volume after two-thirds partial hepatectomy (PH) in approximately 10 days [8, 9]. Serial transplantation experiments have shown that hepatocyte can divide at least 69 times, demonstrating the clonogenic potential of hepatocytes—one of the crucial properties of an SC [10]. In HCV infected liver, the hepatocyte proliferation rate increases with increasing cellular damage [11]. Many models of liver cancer utilize a brief exposure to a genotoxic carcinogen at a time when the liver is in a proliferative state, either during the period after a PH or necrogenic insult [12]. Hepatocytes have been found to be directly involved in carcinogenesis of HCC in 2-acetylaminflourene and DEN-treated rats where hepatocytes were labeled with *β*-galactosidase [13]. Hepatocytes in proliferation appear to be the origin of cancer.

### 2.2. Oval/Liver Progenitor Cells as Targets for Malignant Transformation

When hepatocyte and/or cholangiocytes are damaged or inhibited in their proliferation, a potential SC compartment located within the smallest branches of the intrahepatic biliary tree, the ductules, and canals of Hering gets activated [14]. The “oval cells” in rodent or “the liver progenitor cells” in human liver involve a population of cells that are bipotential and capable of differentiating into hepatocytes or cholangiocytes. The oval/progenitor cells are labeled by over 30 surface markers including biliary-type cytokeratin (CK), CK7, CK19, oval cell markers OV6 and OV1, neuroendocrine marker chromogranin A, neural cell adhesion molecule and parathyroid hormone-related peptide, and connexin 43. The origin of HCC from hepatic progenitor cells (HPC) is is often suggested from the fact that tumors contain an admixture of mature cells and cells phenotypically similar to HPCs [15, 16]. Oval/HPC proliferations and activations are observed after severe liver parenchyma injury, viral hepatitis, alcoholic hepatitis, and nonalcoholic fatty liver disease. HPC/oval cell activation accompanies many instances of liver damage, irrespective of etiology, suggesting such cells are carcinogen targets during hepatocarcinogenesis. Oval cells from p53-null mice formed HCC when transplanted into athymic nude mice [17]. A probable origin from oval cells is suggested by the fact that if oval cell expansion is blocked in the CDE diet mouse modeling by targeting c-Kit with imatinib mesylate, the HCC formation is reduced [18]. Furthermore, the gene expression profile from a selected group of HCC is consistent with the profile of HPCs.

### 2.3. Bone Marrow-Derived Stem Cells

Petersen et al. demonstrated that hepatocytes could be derived from circulating bone marrow cells [19]. Hematopoietic Stem Cells (HSC) from wild-type mice were able to repopulate the liver of FAH-deficient (fah^−/−^) mice [20]. In the setting of sex-mismatched bone marrow transplantation, bone marrow-derived hepatocyte are found in the recipient liver with a large variation in their frequency ranging from less than 1% to >40%. However, in a chimerical mouse model with genetically labeled bone marrow, there was no malignant transformation of the bone marrow-derived liver SC during hepatocarcinogenesis induced by chemical carcinogen [20]. These results suggest that bone marrow-derived liver SC may not be targets for malignant transformation in HCC.

### 2.4. Isolation of Liver Cancer Stem Cells

In the last decade, identification of specific cell surface markers for each of the liver cell type, production of corresponding monoclonal antibodies, and cell sorting techniques have together revolutionized the characteristics of normal stem cells. It has been show that cancer SCs in HCC can be identified by several cell surface antigen CD133, CD90, CD44, OV6, and epithelial cell adhesive molecule (EpCAM), or by selecting the side population (SP) cells in Hoechst dye-staining [21–25].  Table 1 shows markers that are associated with liver cancer SC. The surface markers enrich HCC cells with greater tumorigenicity in immunodeficient mice, higher colon-forming efficiency, and proliferation ability in vitro. In addition, most of the markers are found to be expressed in only a minute proportion of HCC cells, and the expression of the markers correlate with poor prognosis and tumor recurrence. However, it remains to be seen how much overlap there is between these various markers, or whether there is a “one-fits-all” marker for cancer SCs in HCC. Most of the markers which are used for isolating cancer SCs from primary tumor samples were established and adapted from established cancer cell lines. It is not clear whether the cancer SCs that are derived from established cancer cell lines and cultured in vitro reflect the SCs from primary tumor in the gene expression of these surface marker. It remains a challenge to isolate enough clonally derived cancer SCs from primary tumor without in vitro propagation for lineage tracking and differentiation experiments, identification of deregulated signaling pathways that lead to the malignant transformation of normal adult SC to cancer SCs.

### 2.5. Pathways Important for Stem Cell Function Are Deregulated in Hepatocarcinogenesis

From HCC animal model and gene array analysis, a growing body of research suggests that many signaling pathways known to be involved in SC maintenance, self-renewal, and pluripotency, are altered in HCC. This alteration may result in the malignant transformation of liver SC [26]. These observations support the hypothesis that molecular changes in HCC originate in cancer SC [27]. Moreover, these pathways could serve as prognostic markers and targets for therapeutic interventions [27].

## 3. WNT/*β*-Catenin

Disrupted Wnt signaling is observed in approximately one-third of all HCC which underscores its importance in carcinogenesis [28]. The Wnt pathway has a fundamental role in embryogenesis with signaling effects on proliferation and apoptosis in developing cells [29]. Wnt pathway activation is essential for maintenance of SC compartment and regulates cellular differentiation [30]. The “canonical Wnt pathway” describes a cascade of events beginning with the translocation of *β*-catenin from the cell membrane into nucleus, where *β*-catenin then acts as a coactivator of the TCF/LEF family of transcription factors, these in turn regulate specific target genes including c-myc, cyclin D1, and survivin [31]. The signaling cascade is normally initiated when Wnt ligand binds to Frizzled (FZD), a transmembrane receptor [32]. FZD then signals to *β*-catenin to escape its association with E-cadherin. The cytoplasmic elements of the activated Wnt pathway prevent *β*-catenin from being phosphorylated by a degradation complex made up of a serine-threonine kinase, GSK3B, protein scaffolds, AXIN, and adenomatous polyposis coli (APC) [29, 31, 32]. Mutations of proteins that may allow *β*-catenin accumulation in the nucleus to promote transcription of its target genes are found in many cancers [33]. Mutation of *β*-catenin, described in HCC, is located in exon 3 of the CTNNB1 gene, which is the phosphorylation site for GSK3B, AXIN1, and AXIN2 mutation. Activation of Wnt signaling has also been demonstrated in different prospectively isolated SC [34]. 20 to 40% of human HCC bear abnormal cytoplasmic and nuclear accumulation of *β*-catenin by immunohistochemical staining [35]. Markers for elevating expression of Wnt include CD 133^+^ and EpCAM^+^ [36]. The knockdown of the expression of EpCAM, a Wnt/beta-catenin signaling target, in liver cancer SC resulted in decreased proliferation, colony formation, migration, and drug resistance [36]. RNA interference machinery- (RNAi-) mediated knockdown of *β*-catenin resulted in the inhibition of lung cancer SCs [34].

## 4. Transformation Growth Factor-Beta (TGF-*β*)

A wide range of secreted factors regulate SC proliferation and fate. TGF-*β* shows remarkable functional conservation between species and between tissues that self-renew through asymmetric divisions or populational asymmetry. TGF-*β* signaling is important for embryonic hepatocyte proliferation, as well as in the formation of gastrointestinal cancer [37–39]. Tang et al. demonstrated that lack of responsiveness to TGF-*β* pathway in liver SC led to carcinogenesis [40]. Subsequently, it has been shown that targeting this pathway using indirect modulation of IL6/STAT3 appeared to be effective in eradication of cancer SC [26, 40]. 

## 5. Hedgehog

The Hedgehog signaling pathway consists of a complex suite of molecules which regulate cell differentiation, regeneration, and stem cell biology. The pathway plays important roles in the development and homeostasis of the gut tissue [41]. Studies have identified a possible role for this pathway in HCC with expression of Sonic, the predominant ligand of the Hedgehog pathway in liver, that is present in up to 60% of human HCC samples [42, 43]. The Hedgehog pathway is deregulated in hepatocarcinogenesis [42]. Genes involved in the Hedgehog pathway are highly expressed in tumorigenic CD133^+^ liver cancer SC [21]. Suppression of Hedgehog pathway not only decreased HCC cell proliferation but also chemosensitized HCC cells to 5-fluorouracil and to the induction of cell apoptosis [44].

## 6. Target CSCs in the Treatment of HCC

Cancer SCs are predicted to mediate tumor recurrence after chemo- and radiation-therapy due to the relative inability of these modalities to effectively target cancer SCs. Eradicating Cancer SCs brings the hope for cure. Interesting results have been demonstrated in inhibiting breast cancer SC by targeting TGF-*β* and Notch pathways [45]. Similarities between normal and malignant SC, at the levels of cell-surface proteins, molecular pathways, cell cycle quiescence, and microRNA signaling present challenges in developing cancer SC-specific therapeutics. Treatment against cancer SCs should be developed targeting known stem cell regulatory pathways that are deregulated in cancer SCs compared to normal SC, as well as through unbiased high-throughput siRNA or small molecule screening. Both experimental approaches require identification and characterization of the putative liver cancer SC in order to target liver cancer SC specifically to decrease the toxicities. The current strategies of identifying cancer SCs are based on the expression of extracellular markers, the growth of cancer SCs in tumor sphere assays under nondifferentiating conditions, dye exclusion due to the overexpression of drug efflux pumps in cancer SCs, and greater tumorigenicity in immunodeficient mice compared cancer cells that are not cancer stem cells. Despite the amount of literature on liver cancer SCs, it is still not clear as to what constitutes a universal liver cancer SC-specific profile. Given the fact that the diverse etiology for hepatocarcinogenesis and multiple types of progenitor cells are involved in malignant transformation, it is unlikely that a universal liver cancer SC-specific profile will be used for therapeutics development. Ultimately, targeting liver cancer SCs in treating HCC will be in the context of personalized medicine.

## 7. Concluding Remark

Current research supports that HCC derived from malignant transformation of HPC. There may be at least three distinct cell lineages with progenitor cell properties susceptible to neoplastic transformation: hepatocyte, oval/hepatic progenitor cells, and bone marrow-derived stem cells. Multiple signaling transduction pathways important for stem cell proliferation and differentiations are found deregulated during hepatocarcinogenesis. Strategies are being developed to identify and characterize the liver cancer SCs. Targeting liver cancer SCs may bring hope in curing HCC.

## Figures and Tables

**Table 1:** Markers that have aided in the identification of stem cells.

Markers
Cluster of differentiation (CD)133+
CD44+
CD45−
CD90^+^
CD34
OV6
Side population (SP)
Epithelial cell adhesion molecule (EpCAM)
OC.2, OC.3, OC.4, OC.5, OC.10
BDS7
Thy-1
c-kit
ABCG2/BCRP1(breast cancer resistance protein)
Connexin 43
Tumor rejection antigen 1-81 (TRA-1-81)
TRA-1-60
Sry-box containing gene 2 (SOX2)
Surface antigen stage-specific embryonic antigen 3 (SSEA-3)
CK7, CK19, CK14
*α*-fetoprotein (AFP)
*γ*-glutamyltranspeptidase
Placental form of glutathione-S-transferase
Flt-3 ligand
DMBT1(deleted in malignant brain tumor 1)
Neural cell adhesion molecule 1(NCAM)/CD56
Chromogranin A
Parathyroid hormone related peptide (PTHrP)
